# Internet Usage, Social Participation, and Depression Symptoms Among Middle-Aged and Older Adult Chinese Individuals Before and During the COVID-19 Pandemic: Evidence From the China Health and Retirement Longitudinal Observational Study

**DOI:** 10.2196/67039

**Published:** 2025-09-22

**Authors:** Siqian Zhang, Shiju Dong, Zexuan Yu, Shuwen Bi, Wen Wen, Jiajia Li

**Affiliations:** 1 Department of Social Medicine and Health Management, Cheeloo College of Medicine, School of Public Health, Shandong University, 44 WenHua West Road, Li'xia District, Jinan, 250012, China, 86 053188382269; 2 NHC Key Lab of Health Economics and Policy Research, Shandong University, Jinan, China; 3 Shandong Provincial Key New Think Tank, Center for Health Management and Policy Research, Shandong University, Jinan, China; 4 The Dartmouth Institute for Health Policy and Clinical Practice, Dartmouth Hitchcock Medical Center, Geisel School of Medicine at Dartmouth, Lebanon, NH, United States

**Keywords:** internet usage functions, 2-way fixed-effect, mediating effect, gender differences, urban-rural differences

## Abstract

**Background:**

While the relationship between internet usage and depression, and the mediating role of social participation in this association, has garnered significant attention, the COVID-19 pandemic has disrupted traditional forms of social participation. The extent to which this disruption has altered the association remains underexplored.

**Objective:**

This study aims to examine the impact of internet usage on depressive symptoms among middle-aged and older adults and to assess how the mediating role of social participation differs before and during the COVID-19 pandemic.

**Methods:**

Data for this study were sourced from the fourth wave (2018: 15,326 observations) and the fifth wave (2020: 15,758 observations) of the China Health and Retirement Longitudinal Study. A 2-way fixed-effects model incorporating an instrumental variable was employed to investigate the relationship between internet usage and depressive symptoms among middle-aged and older adults. Furthermore, a mediation effect model was applied following propensity score matching to assess changes in the mediating role of social participation on the relationship between internet usage and depressive symptoms before and during the pandemic, as well as to explore changes in 3 internet usage functions. Subgroup analyses based on age and urban-rural residence were performed to assess heterogeneity in this association.

**Results:**

The average Center for Epidemiological Studies Depression Scale (CESD-10) score among middle-aged and older Chinese people increased from 1.94 in 2018 to 1.98 in 2020. Internet usage was negatively associated with depressive symptoms (β=−.041; *P*<.01), with social participation serving as a partial mediator. The proportion of the mediating effect of social participation on the relationship between distinct internet usage functions and depression shifted during the pandemic: the social function declined from 12.55% in 2018 to 9.30% in 2020, while the informational and recreational function increased from 7.53% and 11.29% in 2018 to 8.85% and 16.37% in 2020, respectively. Both the total and indirect effects were statistically significant across subgroups, with a higher mediation proportion observed among rural residents and women than among urban residents and men.

**Conclusions:**

Internet usage was negatively associated with depressive symptoms among middle-aged and older adults. Despite a noticeable increase in depression scores in 2020 compared with 2018, the mediating effect of social participation diminished, while the direct effect of internet usage became more pronounced during the pandemic. These findings underscore the need to prioritize mental health recovery in this population, promote diverse forms of social participation, and leverage the internet’s potential to enhance social participation and mental well-being.

## Introduction

### Background

Depression is now recognized as the predominant psychological issue among older adult populations [1]. From 1994 to 2020, approximately 31.74% of the global older adult population experienced depression, with the incidence rising to 40.78% in low-income countries, significantly higher than the 17.05% observed in high-income nations [1]. The sudden outbreak of the COVID-19 pandemic has rapidly increased the prevalence of depression [2]. The prevalence of depression among American adults surged from 8.5% before the pandemic to 27.8% during its occurrence [3]. Similarly, the prevalence of depression among older adults in Asia and Europe varied from 14.6% to 47.2% during the pandemic [4].

There are several potential factors that may contribute to the increased prevalence of depression during the pandemic, such as difficulty in receiving medical services, particularly mental health services, which has led to reduced social interaction and increased loneliness [5-9]. During this period, the internet has become a vital channel for socialization, kinship supports, and medical consultations, drawing increased attention to its role [10-16]. Previous studies have confirmed the positive impact of the internet on reducing depression in both adults and older individuals [17-19]. However, as a disadvantaged group in internet usage, older adults usually lack basic information literacy [20,21] and are more prone to experiencing digital exclusion [22-25]. For instance, in Australia, while 87% of the population use the internet, only 55% of those aged 65 years and older engage with web-based platforms [26]. Similarly, the internet usage rate of Chinese older adult individuals was merely 13%, far below the 73.8% usage rate among younger groups [27]. The older adults, being a vulnerable population in terms of both health and internet usage, necessitate further research on their mental well-being and the impact of the internet.

### Literature Review

#### Prepandemic Internet Usage, Social Participation, and Depression

##### Internet Usage and Depression

The internet plays an increasingly crucial role in different aspects of people’s lives due to advancements in information technology [28]. As an online platform, it offers convenient access to information, provides entertainment through short videos and games, and facilitates communication among family and friends via email, online chat, and video calls [29].

Academic interest is intensifying in the exploration of how internet usage might influence mental health, with a particular focus on symptoms of depression. Four mechanisms could elucidate the link between internet usage and depression [30-32]. First, internet usage influences depression by sustaining and broadening the social networks of older adults, thereby reducing social isolation and improving the quality of their interpersonal relationships [33,34]. Second, communication through the internet has been found to help older adults strengthen connections with family and friends and gain informal social support [35,36], leading to reduced levels of depression and increased life satisfaction [37]. Third, internet usage has also demonstrated its usefulness in helping middle-aged and older individuals obtain health-related information, increasing their health literacy and modifying behaviors, resulting in a decrease in the occurrence of chronic diseases [38,39]. Finally, the internet provides a wide range of entertaining activities that can bring pleasure to middle-aged and older adult individuals, fulfilling their emotional needs, alleviating symptoms of depression, and enhancing cognitive functions [32,40]. Conversely, some studies have reported that increased time spent on the internet correlates with higher levels of depressive symptoms [41,42]. Specifically, excessive internet usage can result in decreased engagement in other activities and a smaller social network, fostering feelings of social isolation, loneliness, and depression [43-45].

##### Social Participation and Depression

Social participation refers to the process of building connections with others by engaging in social or community activities [46]. Previous studies have demonstrated that active participation in social activities is often associated with improved mental health outcomes [47,48]. In particular, social participation has been found to have a protective effect in relieving depression among older adults through emotional social support and increasing cognitive demands [47,49,50].

However, with aging and the resultant changes in social roles, middle-aged and older adult individuals may face difficulties in maintaining their current interpersonal relationships and social networks [51]. Older adults are more susceptible to feelings of isolation and may experience poorer mental health due to limited social engagement, including factors such as lack of social connections, smaller social networks, or reduced social interaction [52]. Interestingly, individuals with limited social ties often derive significant benefits from formal social engagements, such as participating in volunteer or charity work, or attending educational or training courses, which can lead to reductions in depressive symptoms and improvement in life quality [53].

##### Internet Usage and Social Participation

The internet, unconstrained by time and space, enables individuals to establish connections with the external world. Using online platforms such as WeChat and Weibo, older adults can exchange information, share daily life events, and discover clubs, volunteer activities, and community organizations [54]. As a vital way for older adults to engage in offline activities, the internet facilitates the establishment of new social connections, cultivates a sense of belonging, and ultimately contributes to enhancing their mental health [55,56].

### Internet Usage, Social Participation, and Depression During the Pandemic

#### Internet Usage During the Pandemic

In efforts to minimize the spread of COVID-19 pandemic, individuals were advised to stay at home and maintain social distancing [57]. The decrease in face-to-face contact led to a significant uptick in the amount of time the public spent on internet usage, particularly on social media [15]. A study on Chinese adults revealed a substantial increase in internet usage, with 72% of participants indicating a growing dependence on online connectivity [58]. Social media usage among adults increased significantly, with weekly social media usage increasing from 17.2 hours to 21.4 hours [59]. Similarly, the older adults reported comparable increases in their use of digital platforms. A study of older adults aged 60 years and older in the United States found that they have significantly increased their use of social media and spent more time on their computers and tablets [60].

#### Internet Usage and Depression During the Pandemic

During the COVID-19 pandemic, older adults turned to online chat as a substitute for face-to-face communication with family members who were not living together [61]. This increased internet usage has led to more frequent contact between older adults and their children, reducing social loneliness and perceived stress levels, thus positively impacting their mental health [62,63]. Additionally, older adults have more time to use the internet for leisure activities related to their hobbies and interests, which can mitigate the negative impacts of the pandemic and enhance their well-being [64].

It is critical to acknowledge that social media can spread misinformation, alarmist content, and exaggerated narratives, potentially fostering fear, anxiety, and depression across the broader population [65]. Individuals who excessively relied on social media for COVID-19 information and continuously focused on pandemic updates may experience higher levels of anxiety and depression symptoms during the pandemic [66-69].

#### Social Participation and Depression During the Pandemic

To prevent the spread of the COVID-19 pandemic, individuals were urged to minimize outdoor activities. A global survey showed a dramatic decrease in adults’ engagement in social activities with family, friends, neighbors, or through recreation, noting a 71.15% drop in total social participation scores as a result of COVID-19 home confinement [70]. In the United States, older adults experienced significant reductions in social interactions and engagements with their families and friends, with 1 out of every 4 older individuals reporting fewer than 1 in-person interaction per week with family and friends [71,72]. The Japanese older adults reported a decrease in both the extent and intensity of community participation [73]. Pandemic-related social distancing measures reduced older adults’ social participation in community groups and family activities, leading to a decline in physical activity and increased feelings of loneliness, which negatively affected their physical and mental health [4,8,74,75].

### Study Objectives

This study aims to explore changes in the impact of internet usage on depression among middle-aged and older adults before and during the pandemic. This study further explores whether the mediating effect of social participation changed during the pandemic. Finally, it examines how social participation plays a role in the relationship between different internet usage functions and depressive symptoms and conducts subgroup analyses by urban or rural residence and gender.

## Methods

### Sample and Data Collection

This study employs data from the China Health and Retirement Longitudinal Study (CHARLS). CHARLS uses a probability proportional to size sampling method, systematically selecting samples from counties, villages, residential areas, households, and individuals. The CHARLS baseline survey covered 150 counties or districts and 450 villages or urban communities across the country, involving 10,257 households and 17,708 individuals, offering comprehensive representation of the middle-aged and older adult population in China. CHARLS respondents are followed every 2 years, using a face-to-face computer-assisted personal interview [76]. At present, CHARLS has conducted 5 waves of surveys (2011, 2013, 2015, 2018, and 2020). For each respondent interviewed in the baseline survey, CHARLS will follow up with them in each subsequent survey. The primary respondents and their spouses from the baseline survey will be tracked throughout the entire life cycle of CHARLS. The response rate for the panel sample has consistently remained above 86% in all rounds of follow-up surveys. Specifically, the repeat participation rates for the 2018 and 2020 surveys were 86.46% and 86.81%, respectively [77]. From late 2019 to early 2020, China experienced the COVID-19 pandemic, which led to a reduction in traditional forms of social participation. The fifth wave of CHARLS was conducted from July to September 2020. During the routine prevention and control phase of the COVID-19 pandemic, it is possible to observe changes in internet usage, social participation, and depressive symptoms among Chinese middle-aged and older adults. Therefore, we used data from the 2018 and 2020 surveys to compare changes in the mediating role of social participation in the relationship between internet usage and depression among middle-aged and older adults before and during the pandemic.

The data-cleaning process proceeded as follows: (1) the data from the 2 waves were matched and merged, resulting in a total of 39,084 observations; (2) cases with missing values for Center for Epidemiological Studies Depression Scale (CESD-10) and internet usage were excluded, totaling 6978 observations; and (3) an additional 1022 cases with missing values in control variables were removed. A total of 15,326 respondents in 2018 and 15,758 respondents in 2020 were selected, with 12,549 respondents participating repeatedly. We used data from the 2018 and 2020 waves to construct the panel dataset, encompassing a total of 31,390 observations.

### Ethical Considerations

Prior to the survey, each participant received a written informed consent form outlining the purpose of the study. In accordance with the Declaration of Helsinki, all CHARLS surveys obtained ethical approval from the Institutional Review Board of Peking University (IRB00001052-11015). The survey content is strictly confidential, and all data about respondents are protected by data security and privacy laws. Images and supplementary materials ensure participant anonymity, with no identifiable information included.

### Variables

#### Depression Symptoms

The dependent variable in this study was depression symptoms, measured using the CESD-10 scores. This scale consists of 10 items and has good validity in measuring emotional and physical distress [78]. The Chinese version of the CESD Scale used in CHARLS demonstrated good reliability and validity, with Cronbach α value reaching 0.815 [79]. In this study, the CESD-10 demonstrated good internal consistency, with a Cronbach α value of 0.800, and sampling adequacy, with a Kaiser-Meyer-Olkin value of 0.881. This scale comprises 8 negative questions and 2 positive questions, each with 4 possible responses. For the negative questions, respondents were scored on a scale of 0‐3 based on the frequency of their symptoms: “rarely or none of the time (<1 day), some or a little of the time (1‐2 days), occasionally or a moderate amount of the time (3‐4 days), and most or all of the time (5‐7 days).” The positive questions were scored in the opposite direction. A cumulative score ranging from 0 to 30 was computed, where higher scores signify elevated levels of depressive symptoms.

#### Internet Usage

In this study, the primary explanatory variable was internet usage. In the CHARLS questionnaire, participants were asked: “Have you used the Internet in the past month,” with responses coded as 1 for “yes” and 0 for “no.” Internet usage functions were measured using the question, “What do you usually do on the Internet?” Referring to the studies by Jiang and Luo [80] and Du et al [81], internet usage was divided into 3 distinct functions: social internet usage (eg, chatting), informational internet usage (eg, watching news and managing finances), and recreational internet usage (eg, watching videos and playing games).

#### Social Participation

This study used social participation as a mediating variable, which was assessed using 6 types of activities from the CHARLS questionnaire [82,83]. The selected activities included (1) visiting and socializing with friends; (2) playing mahjong, chess, cards, and engaging in community room activities; (3) providing help to noncohabiting relatives, friends, or neighbors; (4) attending social, sports, or other kinds of clubs; (5) involving in community organizations; and (6) volunteering, engaging in charity work, or caring for sick or disabled individuals not living with the respondent. Referring to the study by Du et al [84] and Yang et al [85], each activity was assigned 1 point, resulting in a total score ranging from 0 to 6, with higher scores indicating higher levels of social participation among respondents.

#### Control Variables

Based on previous studies [84,86-89], we included several control variables in the analysis that have been associated with depressive symptoms in the CHARLS dataset. These variables included gender (man=0, woman=1), age (continuous variable), residence (urban=0, rural=1), education (illiterate=0, primary school or below=1, middle school=2, and high school or above=3), marital status (others=0, married=1), chronic diseases (none=0, 1 type=1, and two or more types=2), sleep duration, nap duration, physical activity, drinking habits (no=0, yes=1), annual per capita consumption expenditure, household size (continuous variable), household composition (living alone=0, living with spouse only=1, and others=2), health care accessibility (no=0, yes=1), and employment status (unemployed=0, employed=1). Marital status was categorized as “others,” which includes those who are separated, divorced, widowed, or never married. Sleep duration was divided into 5 groups:<6 hours, 6‐7 hours, 7‐8 hours, 8‐9 hours, and ≥9 hours per night [90]. Nap duration was categorized into 4 groups: nonnappers (0 minutes), short nappers (<30 minutes), moderate nappers (30‐90 minutes), and excessive nappers (>90 minutes) [91]. Physical activity was measured based on intensity of activity performed each week and was classified into 3 groups: light, moderate, and intensive physical activity [92]. Health care accessibility was defined based on the concept of the “15-minute medical circle,” referring to the ability of residents to access health care services within a 15-minute walk or via convenient transportation.

### Data Analysis

#### Two-Way Fixed-Effects and Instrumental Variables Model

Two-way fixed-effects (TWFE) model is a panel data method that accounts for both individual and time fixed effects, thereby controlling for unobserved confounders that are constant across time or individuals. Given that the CESD-10 score used in this study was a continuous variable, we employed a TWFE model to examine the impact of internet usage and social participation on depressive symptoms over time. Furthermore, given the potential endogeneity issues, we used smartphone usage as an instrumental variable for internet usage for middle-aged and older adults. The instrumental variable–fixed-effects (IV-FE) model was used to estimate the impact of internet usage on depression symptoms.

In the first stage, the instrumental variable (smartphone usage) was used to predict the endogenous variable, internet usage, yielding the fitted values, ${Internet\_use}_{it}\overset{OLS}{\to }{IV}_{it}$, to obtain the fitted values $\hat{{Internet\_use}_{it}}$. In the second stage, the outcome variable, depressive symptoms, was regressed on the fitted internet usage values obtained from the first stage. The premise of using IV was the existence of endogenous explanatory variables. In addition, the correlation and validity of IV were tested. The basic model was as follows:


(1) $$Depression_{it}=\beta_{0}Internet\_use_{it}++\beta_{1}XX_{it}+\sigma_{i}+\gamma_{t}+\mu_{it}$$

(2) $${Internet\_use}_{it}=\alpha_{0}Z_{it}+\alpha_{1}T_{it}+\sigma_{i}+\gamma_{t}+\varepsilon_{it}$$

where ${Depression}_{it}$ represents the continuous outcome variable and $Internet\_use_{it}$ is a binary indicator of internet usage. ${XX}_{it}$ denotes a vector of control variables. $Z_{it}$ represents smartphone use, and $T_{it}$ is the exogenous in the equation of ${Depression}_{it}$ variable, $\sigma_{i}$ and $\gamma_{t}$ capture individual and time fixed effects, respectively, while $\mu_{it}$ and $\varepsilon_{it}$ are the error terms.

#### Propensity Score Matching–Based Mediating Effect Model

To address potential selection bias arising from the non-random nature of internet usage, we implemented propensity score matching (PSM) prior to mediating effect analysis [93,94]. Propensity scores were estimated using k-nearest neighbor matching, radius matching, and kernel-matching methods. The following covariates were included in the PSM adjustment: gender, age, residence, education, marital status, presence of chronic disease, sleep duration, nap duration, physical activity, drinking behavior, per capita household expenditure, household size and composition, health care accessibility, and employment status. To explore the mechanism underlying the impact of internet usage on depression among middle-aged and older individuals, a mediating effect model was constructed following the approach of Wen et al [95]. The models were set as follows:


(3) $$Social\_participation_{it}=\alpha_{2}+\beta_{2}Internet\_use_{it}+\beta_{3}X_{it}+\delta_{it}$$

(4) $$\begin{array}{l} {Depression}_{it}=\alpha_{3}+\beta_{4}\;Interne\_use_{it}+\\ \beta_{5}\;{Social\_participation}_{it}+\beta_{6}\;X_{it}+\varphi_{it} \end{array}$$

where $\beta_{2}$ represents the regression coefficient of internet usage on mediating variable, and $\beta_{3}$ denotes the regression coefficient of internet usage on CESD-10 score among middle-aged and older individuals after adding mediating variable. Mediation analysis was conducted using the *sgmediation* package in Stata (version 14.0; StataCorp), with 5000 bootstrap replications to validate the mediating effect of social participation before and during the COVID-19 pandemic [96]. This allowed us to estimate the mediating effect of social participation and obtain bias-corrected 95% CIs for the indirect, direct, and total effects. The root-mean-square error of approximation for the mediation model was 0.000, which is below the cutoff value of 0.08, indicating excellent model fit. Moreover, we examined the proportion of the mediating effect of social participation between different internet usage functions and depression symptoms.

## Results

### Descriptive Statistics

**Figure 1.**
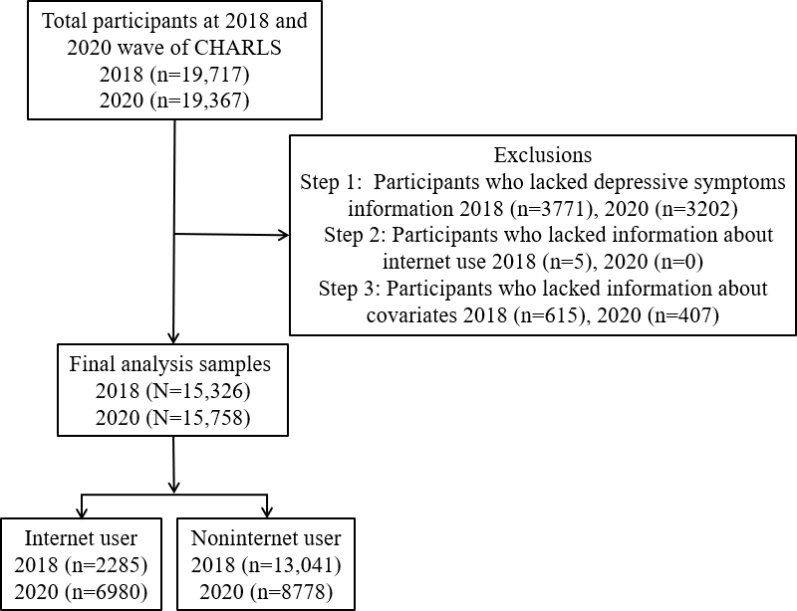
Flowchart of the study population selection process. CHARLS: China Health and Retirement Longitudinal Study.

Figure 1 shows the flowchart of the study population selection process. Table S1 in Multimedia Appendix 1 shows the participant characteristics at each wave of data collection. The results indicated an increase in depression symptom scores in 2020 compared with 2018. CESD-10 scores rose from 1.94 (SD 0.86) to 1.98 (SD 0.84). Social participation scores dropped from 0.81 (SD 0.99) in 2018 to 0.79 (SD 1.00) in 2020. In 2018, the majority of respondents were women (7820/15,326, 51.02%), with 70.59% residing in rural areas and 87.75% being married. Of the respondents in 2020, the majority lived in rural areas (9967/15,758, 63.25%) and most of them were women (8162/15,758, 51.80%). A large proportion had a primary school education or below (6886/15,758, 43.70%). The majority of respondents were married ( 13,599/15,758, 86.30%).

### Two-Way Fixed-Effects Regression Results

The results of the TWFE model are shown in Table 1. As shown, both internet usage (β=−.041; *P*<.01) and social participation (β=−.016; *P*<.05) have a negative impact on depression symptoms. Additionally, depression symptoms worsened between 2018 and 2020 (β=.060; *P*<.001).

**Table 1.** Results of regressions with 2-way fixed effects (N=31,084).

Variable	β coefficient	SE	*P* value
Internet usage (reference: no)	−0.041	0.015	.008
Social participation	−0.016	0.007	.02
Residence (reference: urban)	0.012	0.020	.06
Marital status (reference: Others)	−0.171	0.049	.001
Chronic diseases (reference: None)			
1 type	0.018	0.013	.16
≥2 types	0.106	0.017	<.001
Sleep duration (hours), reference: <6			
6-7	−0.091	0.016	<.001
7-8	−0.123	0.019	<.001
8-9	−0.163	0.020	<.001
≥9	−0.128	0.027	<.001
Nap duration (reference: nonnappers)			
Short nappers	−0.020	0.017	.39
Moderate nappers	−0.004	0.016	.81
Excessive nappers	−0.017	0.022	.44
Physical activity (reference: light)			
Moderate	−0.008	0.016	.61
Intensive	−0.005	0.016	.77
Drinking (reference: no)	0.022	0.008	.01
Household expenditure per capita	−0.009	0.007	.03
Household size	−0.007	0.005	.26
Household composition (reference: living alone)			
Living with spouse only	0.007	0.035	.84
Others	0.025	0.039	.53
Employment status (reference: no)	−0.035	0.019	.07
Year (reference: 2018)	0.048	0.010	<.001

### IV-FE Regression Results

Table 2 reports the results of the IV-FE regression. The coefficient of internet usage on depression is −0.044, statistically significant at the 1% level. The Kleibergen-Paap rk LM statistic and the Cragg-Donald Wald F statistic confirm the validity of the instruments [97]. This indicates that the negative impact of internet usage on depression among middle-aged and older adults remains robust after controlling for sources of endogeneity.

**Table 2.** Result of the instrumental variable–fixed-effects method (N=25098).

Variable	β coefficient	SE	*P* value
Internet usage (reference: no)	−0.044	0.016	.006
Social participation	−0.016	0.007	.02
Residence (reference: rural)	0.012	0.021	.56
Marital status (reference: married)	0.171	0.049	<.001
Chronic diseases (reference: ≥2 types)			
None	−0.106	0.015	<.001
One type	−0.089	0.017	<.001
Sleep duration (hours), reference:≥9			
<6	0.128	0.028	<.001
6-7	0.037	0.028	.18
7-8	0.005	0.028	.86
8-9	−0.034	0.028	.21
Nap duration (reference: excessive nappers)			
Nonnappers	0.017	0.023	.45
Short nappers	−0.003	0.027	.91
Moderate nappers	0.013	0.021	.52
Physical activity (reference: intensive)			
Light	0.005	0.016	.77
Moderate	−0.003	0.014	.83
Drinking (reference: yes)	−0.022	0.008	.01
Household expenditure per capita	−0.009	0.007	.19
Household size	−0.007	0.006	.26
Household composition (reference: others)			
Living alone	−0.025	0.037	.84
Living with spouse only	−0.017	0.019	.51
Employment status (reference: yes)	0.034	0.018	.06
Year (reference: 2020)	−0.049	0.011	<.001
KP-LM^a^	5883.626		
CD-Wald^b^	3.0e+05		

a KP-LM: Kleibergen-Paap rk LM statistic, testing the relevance of IV.

b CD-Wald: Cragg-Donald Wald F statistic, testing the weak relevance of IV.

### Mediating Effects After PSM Analysis

**Figure 2.**
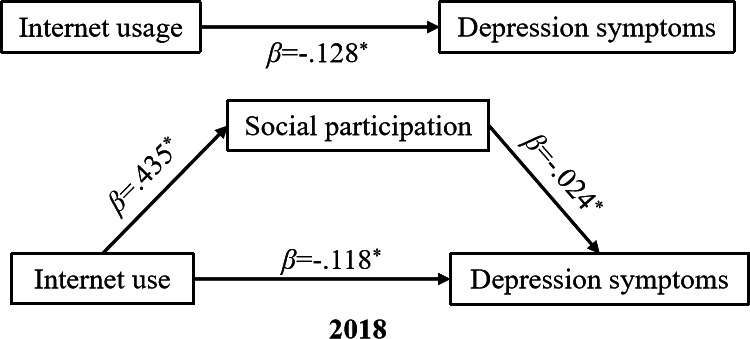
Path diagram of the impact of internet usage on depression symptoms in 2018. The model controls for residence, marital status, chronic diseases, sleep duration, nap duration, physical activity, drinking, household expenditure per capita, household size, household composition, and employment status. ^*^P<.001.

Figures2  3 and Table 3 illustrate the PSM-based indirect effects examined between internet usage and depression before and during the COVID-19 pandemic. The findings demonstrated that social participation significantly (*P*<.001) played a role in mediating the relationship between internet usage and depression symptoms both in 2018 and 2020. Particularly, internet usage among middle-aged and older adults led to a notable increase in their level of social participation (2018, β=.435, *P*<.001; 2020, β=.336, *P*<.001), indicating a moderate to strong standardized effect size. Significant direct effects (2018, β=−.118, *P*<.001; 2020, β=−.095, *P*<.001) as well as indirect effects (2018, β= −.010, *P*<.01; 2020, β=−.011, *P*<.001) of internet usage on depression were observed among the middle-aged and older groups. The coefficient reflecting the impact of internet usage on depression decreased when social participation was included as a mediator. With the inclusion of social participation, the coefficient changed from −0.128 (*P*<.001) to −0.118 (*P*<.001) in 2018, and from −0.106 (*P*< .001) to −0.095 (*P*<.001) in 2020.

The balance test results are shown in Tables S2-S4 in Multimedia Appendix 1. After matching, the standardized differences of covariates were substantially reduced, and no significant differences were observed between the treatment and control groups. These results indicate that systematic differences between groups were largely eliminated.

Table S5 in Multimedia Appendix 1 shows the mediation analysis results using 3 different matching methods: k-nearest neighbor, radius, and kernel matching. The findings consistently support the main results, demonstrating a robust mediating effect of social participation on the relationship between internet usage and depressive symptoms among middle-aged and older Chinese adults.

**Figure 3.**
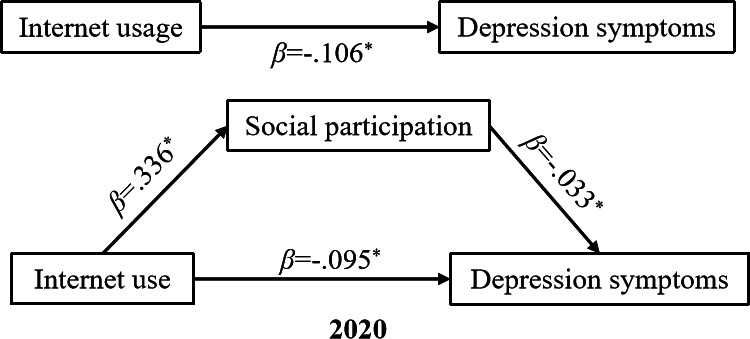
Path diagram of the impact of internet usage on depression symptoms in 2020. The model controls for residence, marital status, chronic diseases, sleep duration, nap duration, physical activity, drinking, household expenditure per capita, household size, household composition, and employment status. ^*^P<.001.

**Table 3.** Mediating effect of social participation between internet usage and depression.

Types	β coefficient (bootstrapped 95% CI)	SE	*P* value
2018			
Indirect effect	−0.010 (−0.018 to −0.003)	0.004	.008
Direct effect	−0.119 (−0.160 to −0.075)	0.022	<.001
Total effect	−0.128 (−0.170 to −0.086)	0.022	<.001
2020			
Indirect effect	−0.011 (−0.016 to −0.006)	0.003	<.001
Direct effect	−0.095 (−0.126 to −0.064)	0.015	<.001
Total effect	−0.106 (−0.137 to −0.076)	0.016	<.001

### Proportion of Mediating Effects in Social Function, Getting Information, and Recreational Function

Table 4 shows the indirect effects of internet usage functions on depression, both prior to and during the COVID-19 pandemic. It reveals that social participation acted as a significant partial mediator in the relationship between different internet usage functions and depression. To be specific, in 2018 and 2020, the indirect effect of social participation on social function and depression was −0.012 (*P*<.01), and −0.010 (*P*<.001), respectively. Similarly, the indirect effects of social participation on getting information and depression were −0.011 (*P*<.01) and −0.011 (*P*<.001), while for recreational function and depression, the indirect effects were −0.012 (*P*<.01) in 2018 and −0.011 (*P*<.001) in 2020. Figure S1 in Multimedia Appendix 1 shows the proportion of mediating effects of social participation between different internet usage functions and depression. It illustrated that in 2018, the mediating effect of social participation between social function and depressive symptoms was more pronounced than in 2020. Conversely, the proportion of the mediating effect of social participation between informational function and depressive symptoms did not significantly change before and during the pandemic.

**Table 4.** The indirect effects of internet usage functions analyzed by pandemic time.

Indirect effects of internet usage analyses by functions	β (bootstrapped 95% CI)	SE	*P* value
2018			
Social function → Social participation → Depression symptoms	−.012 (−0.020 to −0.004)	0.004	.002
Informational function → Social participation → Depression symptoms	−.011 (−0.019 to −0.003)	0.004	.007
Recreational function → Social participation → Depression symptoms	−.012 (−0.019 to −0.004)	0.004	.003
2020			
Social function → Social participation → Depression symptoms	−.010 (−0.015 to −0.005)	0.002	<.001
Informational function → Social participation → Depression symptoms	−.011 (−0.017 to −0.006)	0.003	<.001
Recreational function → Social participation → Depression symptoms	−.011 (−0.016 to −0.006)	0.003	<.001

### Subgroup Analysis

Table 5 presents the mediating effects of social participation across subgroups defined by residence (urban vs rural) and gender (man vs woman). Social participation significantly mediated the relationship between internet usage and depressive symptoms (*P*<.001). Specifically, internet usage was associated with reduced depression scores among middle-aged and older adults, with pronounced heterogeneity observed across demographic subgroups.

In 2018, the indirect effect of social participation was not significant among rural residents (β=−.006, 95% CI −0.016 to 0.005) and women (β=−.007, 95% CI −0.017 to 0.004). By 2020, however, these effects became significant in both subgroups—rural: β=−.012 (95% CI −0.019 to −0.005); woman: β=−.011 (95% CI −0.018 to −0.005)—with higher mediation proportions observed compared with urban residents and men. The direct effects of internet usage on depression also showed consistent negative associations across all groups, with rural residents and women exhibiting slightly stronger direct effects in 2018. By 2020, direct effects converged across subgroups, while the emergence of significant indirect effects in rural groups and women highlighted the growing role of social participation as a mediator. The total effect of social participation on the relationship between internet usage and depressive symptoms was greater among rural residents and women, both before and during the pandemic.

**Table 5.** Subgroup analysis of the mediating effect of social participation between internet usage and depressive symptoms.

Types	Residence		Gender	
	Urban, β (bootstrapped 95% CI)	Rural, β (bootstrapped 95% CI)	Man, β (bootstrapped 95% CI)	Woman, β (bootstrapped 95% CI)
2018				
Indirect effect	−.014^a^ (−0.025 to −0.002)	−.006 (−0.016 to −0.005)	−.012^a^ (−0.024 to −0.001)	−.007 (−0.017 to 0.004)
Direct effect	−.090^a^ (−0.151 to −0.028)	−.146^b^ (−0.205 to −0.087)	−.110^b^ (−0.169 to −0.052)	−.127^b^ (−0.189 to −0.066)
Total effect	−.103^b^ (−0.164 to −0.042)	−.151^b^ (−0.210 to −0.092)	−.123^b^ (−0.181 to −0.065)	−.134^b^ (−0.196 to −0.073)
2020				
Indirect effect	−.008^c^ (−0.017 to 0.001)	−.012^b^ (−0.019 to −0.005)	−.009^a^ (−0.017 to −0.002)	−.011^a^ (−0.018 to −0.005)
Direct effect	−.089^b^ (−0.142 to −0.036)	−.093^b^ (−0.132 to −0.054)	−.103^b^ (−0.153 to −0.053)	−.102^b^ (−0.146 to −0.058)
Total effect	−.097^b^ (−0.149 to −0.045)	−.105^b^ (−0.144 to −0.066)	−.112^b^ (−0.162 to −0.063)	−.113^b^ (−0.157 to −0.069)

a *P*<.05.

b *P*<.001.

c *P*<.01.

## Discussion

### Principal Findings

Using data from the CHARLS and applying the IV-FE method, this study found that internet usage was negatively associated with depressive symptoms among middle-aged and older adults. Social participation partially mediated this relationship. Although depression scores increased in 2020 compared with 2018, the mediating effect of social participation weakened, while the direct effect of internet was strengthened during the pandemic. Additionally, the proportion of the mediating effect of social participation on the relationship between internet usage functions and depressive symptoms shifted over time. Specifically, the social function declined from 12.55% in 2018 to 9.30% in 2020, whereas the informational and recreational functions increased from 7.53% and 11.29% in 2018 to 8.85% and 16.37% in 2020, respectively.

Findings from the study indicate an increase in the average CESD-10 score among middle-aged and older Chinese individuals, rising from 1.94 in 2018 to 1.98 in 2020. This upward trend was less pronounced compared with other countries during the same time frame [98-100]. The prevalence of milder or greater depressive symptoms increased by 1.6 times among the older adults with diabetes in the United States. In the United Kingdom, the prevalence of mental health problems in adults rose from 24.3% in 2017‐2019 to 37.8% in April 2020. Additionally, the prevalence of depression among older adults in Ireland surged from 7.2% before the pandemic to 19.8% during the pandemic. Depressive symptoms in China during the COVID-19 pandemic were less severe than in other countries, primarily due to China’s web-based mental health services and community support [101,102].

This study suggests that internet usage had a significant reduction of 4.10% in depressive symptoms, aligning with findings from previous research [103-106]. Meanwhile, we found that the internet played a mitigating role in the worsening of depressive symptoms among middle-aged and older adults following the outbreak. The reason may be that the internet enables the older adults to access health-related information, monitor health indicators, and enhance communication with family and friends [12,39]. Particularly, the internet serves as a platform for middle-aged and older adults to gather information on social activities, leading to greater engagement in such activities [107,108]. Through internet technology, middle-aged and older adult people can explore new social participation opportunities, assume new social roles, and expand their social networks based on existing interpersonal relationships [109,110]. Notably, our study indicates that web-based social participation may act as a complement to offline engagement, aiding individuals in avoiding social isolation [33,111].

In this study, we also explore the relationship between different internet usage functions, social participation, and depressive symptoms before and during the COVID-19 pandemic. First, in terms of social function, the proportion of the mediating effect of social participation decreased from 12.55% in 2018 to 9.30% in 2020. One explanation is that the social functions of the internet increase enhanced interactions among people and facilitate their engagement in various volunteer activities [112], thereby expanding interpersonal networks, improving the quality of interpersonal relationships, and obtaining more emotional support [34]. Another explanation may be a substitution effect between social functions and social participation, wherein certain offline social activities, such as playing mahjong or card games, were replaced by web-based activities. Second, the mediation effect proportion of social participation between information acquisition function and depressive symptoms had slightly increased before and during the pandemic. One possible reason for this is that people pay more attention to health and pandemic-related information (eg, infection and mortality rates) during the pandemic [69,113]. Finally, concerning recreational functions, the mediating effect of social participation increased during the pandemic. The internet provides a wide range of recreational activities, allowing individuals to choose leisure activities based on their interests [84]. Additionally, during the pandemic, people had ample spare time to partake in web-based recreational activities, such as gaming or watching videos, as a means to alleviate anxiety and depression [114].

Given the urban-rural digital divide and gender-based differences in internet usage patterns [85], stratified analyses revealed that the proportion of mediation was higher among rural residents and women than among urban residents and men. This difference may reflect disparities in infrastructure and access across settings [34]. Despite limitations in rural internet infrastructure, older adults who do access the internet may gain stronger social and psychological benefits. For rural populations, internet usage may serve as a critical tool to overcome reduced mobility and limited social opportunities during the pandemic, thereby fostering social inclusion and emotional support that contribute to reduced depressive symptoms [85]. Gender differences in the mediating role of social participation may also stem from distinct usage patterns and social norms. Women tend to use the internet more for social communication and emotional connection, which may enhance the protective effect of internet usage against depression via increased social participation. Men, on the other hand, may use the internet more for information seeking or entertainment, which might have a less direct impact on social engagement and thus a weaker mediating effect.

### Limitations

Several limitations should also be aware of in this study. First, the study did not perform a comprehensive analysis of the intensity and types of internet usage, including daily time spent on the web, which could impact mental health. Second, reliance on self-reported data for depression and internet usage may introduce biases, such as recall bias or social desirability. Third, reliance on cross-sectional data for mediation analysis in our study limits our capacity to determine a causal relationship between internet usage and depression. Future studies should delve deeper into the causal association between internet usage duration, device preference, and mental health in middle-aged and older adults, and examine the role of different types of social participation.

### Conclusions

Using national survey data, this study examined the mechanisms of internet usage on depression in middle-aged and older adults, across periods both before and during the COVID-19 pandemic. Internet usage was found to mitigate depression levels, with social participation serving as a key mediator between internet usage and depression. Therefore, measures to promote and amplify the positive impact of internet usage among older individuals are vital, such as offering community-based computer training, developing age-friendly web-based apps, and leveraging big data for providing services tailored to the older adults. Considering the influence of digital literacy on the effectiveness of internet usage, targeted training programs for older adults, especially for rural residents and women, should be prioritized. Mental health recovery in aging populations should also be advanced by integrating internet usage facilitation and social engagement strategies into broader public health and social welfare systems.

## Supplementary material

10.2196/67039 Multimedia Appendix 1 Basic descriptive statistics, balance test results for different matching methods, propensity score matching–based mediating effect, and comparison of proportions of mediating effects on the internet usage function.

## Abbreviations

CESD: Center for Epidemiological Studies Depression Scale
CHARLS: China Health and Retirement Longitudinal Study
IV-FE: instrumental variable–fixed effects
PSM: propensity score matching
TWFE: 2-way fixed effects
