# Supplementary material for: Internet Usage, Social Participation, and Depression Symptoms Among Middle-Aged and Older Adult Chinese Individuals Before and During the COVID-19 Pandemic: Evidence From the China Health and Retirement Longitudinal Observational Study
Source: J Med Internet Res. 2025 Sep 22;27:e67039. doi: 10.2196/67039 (PMC12479384; doi:10.2196/67039)
Supplement: Multimedia Appendix 1 [file jmir-v27-e67039-s001.docx]

**Table S1.** Basic descriptive statistics in 2018 (N=15326) and 2020 (15758)^a^.

| **Variable** | 2018 | | | | 2020 | | | | |
| --- | --- | --- | --- | --- | --- | --- | --- | --- | --- |
|  | Total  (N = 15326) | Non-Internet user  (N = 13041) | Internet user  (N = 2285) | *P* value | | Total  (N = 15758) | Non-Internet user  (N = 8778) | Internet user  (N = 6980) | *P* value |
| **Depression score, mean (SD)** | 1.94 (0.86) | 1.99 (0.86) | 1.67 (0.86) | <.001 | | 1.98 (0.84) | 2.11 (0.79) | 1.81 (0.0.87) | <.001 |
| **Social participation, mean (SD)** | 0.81 (0.99) | 0.71 (0.91) | 1.37 (1.24) | <.001 | | 0.79 (1.00) | 0.58 (0.81) | 1.06 (1.14) | <.001 |
| **Gender, n (%)** |  |  |  | <.001 | |  |  |  | <.001 |
| Male | 7506 (48.98) | 6249 (83.25) | 1257 (16.75) |  | | 7596 (48.20) | 4062 (53.48) | 3534 (46.52) |  |
| Female | 7820 (51.02) | 6792 (86.85) | 1028 (13.15) |  | | 8162 (51.80) | 4716 (57.78) | 3446 (42.22) |  |
| **Age, mean (SD)** | 60.80 (9.25) | 61.72 (9.24) | 55.53 (7.41) | <.001 | | 62.41 (9.10) | 66.01 (8.84) | 57.89 (7.22) | <.001 |
| **Residence, n (%)** |  |  |  | <.001 | |  |  |  | <.001 |
| Urban | 4508 (29.41) | 3205 (71.02) | 1303 (28.90) |  | | 5791 (36.75) | 2230 (40.23) | 3461 (59.77) |  |
| Rural | 10818 (70.59) | 9836 (90.92) | 982 (9.08) |  | | 9967 (63.25) | 6448 (64.69) | 3519 (35.31) |  |
| **Education, n (%)** |  |  |  | <.001 | |  |  |  | <.001 |
| Illiterate | 2783 (18.16) | 2746 (98.67) | 37 (1.33) |  | | 2932 (18.61) | 2357 (86.53) | 395 (13.47) |  |
| Primary school or below | 6739 (43.97) | 6199 (91.99) | 540 (8.01) |  | | 6886 (43.70) | 4345 (63.10) | 2541 (36.90) |  |
| Middle school | 3689 (24.07) | 2867 (77.72) | 822 (22.28) |  | | 3779 (23.98) | 1404 (37.15) | 2375 (62.85) |  |
| High school or above | 2115 (13.80) | 1229 (58.11) | 886 (41.89) |  | | 2161 (13.71) | 492 (22.77) | 1669 (77.23) |  |
| **Marital status, n (%)** |  |  |  | <.001 | |  |  |  | <.001 |
| Others | 1878 (12.25) | 1714 (91.27) | 164 (8.73) |  | | 2159 (13.70) | 1588 (73.55) | 571 (26.45) |  |
| Married | 13448 (87.75) | 11327 (84.23) | 2121 (15.77) |  | | 13599 (86.30) | 7190 (52.87) | 6409 (47.13) |  |
| **Chronic diseases, n (%)** |  |  |  | .750 | |  |  |  | .009 |
| None | 8600 (56.11) | 7305 (84.94) | 1295 (15.06) |  | | 10026 (63.62) | 5529 (55.15) | 4497 (44.85) |  |
| One type | 4211 (27.48) | 3593 (85.44) | 613 (14.56) |  | | 3781 (23.99) | 2100 (55.54) | 1681 (44.46) |  |
| Two or more types | 2515 (16.41) | 2138 (85.01) | 377 (14.99) |  | | 1951 (12.38) | 1149 (58.89) | 802 (41.11) |  |
| **Sleep duration** |  |  |  | <.001 | |  |  |  | <.001 |
| < 6 | 5117 (33.39) | 4486 (87.67) | 631 (12.33) |  | | 5567 (35.33) | 3365 (60.45) | 2202 (39.55) |  |
| 6~7 | 3543 (23.12) | 2872 (81.06) | 671 (18.94) |  | | 4053 (25.72) | 1968 (48.56) | 2085 (51.44) |  |
| 7~8 | 2699 (17.61) | 2193 (81.25) | 506 (18.75) |  | | 2733 (17.34) | 1348 (49.32) | 1385 (50.68) |  |
| 8~9 | 2782 (18.15) | 2392 (85.98) | 390 (14.02) |  | | 2443 (15.50) | 1392 (56.98) | 1051 (43.02) |  |
| ≥ 9 | 1185 (7.73) | 1098 (92.66) | 87 (7.34) |  | | 962 (6.10) | 705 (73.28) | 257 (26.72) |  |
| **Nap duration** |  |  |  | <.001 | |  |  |  | <.001 |
| Non-nappers | 5859 (38.23) | 5112 (87.25) | 747 (12.75) |  | | 5644 (35.82) | 3463 (61.36) | 2181 (38.64) |  |
| Short nappers | 1296 (8.46) | 1069 (82.48) | 227 (17.52) |  | | 1038 (6.59) | 464 (44.70) | 574 (55.30) |  |
| Moderate nappers | 6107 (39.85) | 5011 (82.05) | 1096 (17.95) |  | | 7218 (45.81) | 3595 (49.81) | 3623 (50.19) |  |
| Excessive nappers | 2064 (13.47) | 1849 (89.58) | 215 (10.42) |  | | 1858 (11.79) | 1256 (67.60) | 602 (32.40) |  |
| **Physical activity, n (%)** |  |  |  | <.001 | |  |  |  | <.001 |
| Light | 1937 (12.64) | 1778 (91.79) | 159 (8.21) |  | | 6298 (39.97) | 3990 (63.35) | 2308 (36.65) |  |
| Moderate | 3950 (25.77) | 3206 (81.16) | 744 (18.44) |  | | 4152 (26.35) | 1785 (42.99) | 2367 (57.01) |  |
| Intensive | 9439 (61.59) | 8057 (85.36) | 1382 (14.64) |  | | 5308 (33.68) | 3003 (56.57) | 2305 (43.43) |  |
| **Drinking, n (%)** |  |  |  | <.001 | |  |  |  | <.001 |
| No | 9807 (63.99) | 8702 (88.73) | 1105 (11.27) |  | | 5959 (37.82) | 2796 (46.92) | 3163 (53.08) |  |
| Yes | 5519 (36.01) | 4339 (78.62) | 1180 (21.38) |  | | 9799 (62.18) | 5982 (61.05) | 3817 (38.95) |  |
| **Household expenditure per capital, mean (SD)** | 8.67 (1.07) | 8.57 (1.06) | 9.22 (0.94) | <.001 | | 8.86 (0.98) | 8.63 (0.99) | 9.15 (0.88) | <.001 |
| **Household size, mean (SD)** | 2.86 (1.40) | 2.86 (1.44) | 2.83 (1.20) | .005 | | 2.97 (1.57) | 2.89 (1.62) | 3.06 (1.51) | <.001 |
| **Household composition, n (%)** |  |  |  | <.001 | |  |  |  | <.001 |
| Living alone | 609 (3.97) | 548 (89.98) | 61 (10.02) |  | | 1219 (7.74) | 886 (72.68) | 333 (27.32) |  |
| Living with spouse only | 7933 (51.76) | 6813 (85.88) | 1120 (14.12) |  | | 6850 (43.47) | 3987 (58.20) | 2863 (41.80) |  |
| Others | 6784 (44.26) | 5680 (83.73) | 1104 (16.27) |  | | 7689 (48.79) | 3905 (50.79) | 3784 (49.21) |  |
| **Healthcare accessibility, n (%)** |  |  |  | <.001 | |  |  |  | <.001 |
| No | 6687 (43.63) | 5971 (89.29) | 716 (10.71) |  | | 6942 (44.05) | 4251 (61.24) | 2691 (38.76) |  |
| Yes | 8639 (56.37) | 7070 (81.84) | 1569 (18.16) |  | | 8816 (55.95) | 4527 (51.35) | 4289 (48.65) |  |
| **Employment status, n (%)** |  |  |  | .001 | |  |  |  | <.001 |
| Unemployed | 5003 (32.64) | 4324 (86.43) | 679 (13.57) |  | | 5024 (31.88) | 3017 (60.05) | 2007 (39.95) |  |
| Employed | 10323 (67.36) | 8717 (84.44) | 1606 (15.56) |  | | 10734 (68.12) | 5761 (53.67) | 4973 (46.33) |  |

^a^ Regarding the statistical description of variables, we use mean and standard deviation (SD) for continuous values and frequency (n) and percentage for categorical variable.

**Table S2.** Balance test results for K-nearest neighbor matching.

| **Variable** | **2018** | | | | | **2020** | | | | | |
| --- | --- | --- | --- | --- | --- | --- | --- | --- | --- | --- | --- |
|  | Mean | | % bias | T-test | | | Mean | | % bias | T-test | |
|  | Treated | Control |  | t | *P* > \| t \| | | Treated | Control |  | t | *P* > \| t |
| **Gender** | 0.449 | 0.444 | 0.9 | 0.31 | .755 | | 0.491 | 0.483 | 1.6 | 0.94 | .345 |
| **Age** | 55.556 | 55.463 | 1.2 | 0.48 | .633 | | 58.053 | 58.078 | -0.3 | -0.20 | .839 |
| **Residence** | 0.431 | 0.431 | 0.1 | 0.03 | .972 | | 0.513 | 0.505 | 1.7 | 0.94 | .347 |
| **Education** | 2.116 | 2.120 | -0.6 | -0.19 | .848 | | 1.740 | 1.759 | -2.2 | -1.24 | .214 |
| **Marital status** | 0.928 | 0.929 | -0.4 | -0.15 | .878 | | 0.917 | 0.905 | 3.7 | 2.57 | .010 |
| **Chronic disease** | 0.599 | 0.620 | -2.8 | -0.93 | .350 | | 0.471 | 0.518 | -6.7 | -3.90 | <.001 |
| **Sleeping duration** | 1.402 | 1.420 | -1.4 | -0.51 | .609 | | 1.297 | 1.319 | -1.7 | -1.06 | .288 |
| **Napping duration** | 1.341 | 1.335 | 0.5 | 0.18 | .858 | | 1.376 | 1.326 | 4.6 | 2.74 | .006 |
| **Physical activity** | 1.535 | 1.550 | -2.2 | -0.79 | .428 | | 0.997 | 0.943 | 6.3 | 3.72 | <.001 |
| **Drinking** | 0.515 | 0.500 | 3.2 | 1.06 | .289 | | 0.552 | 0.575 | -4.8 | -2.71 | .007 |
| **Household expenditure per capital** | 9.214 | 9.195 | 1.8 | 0.71 | .475 | | 9.127 | 9.119 | 0.8 | 0.55 | .582 |
| **Household size** | 2.830 | 2.843 | -1.0 | -0.37 | .712 | | 3.061 | 3.094 | -2.1 | -1.26 | .209 |
| **Household composition** | 1.455 | 1.462 | -1.2 | -0.41 | .681 | | 1.490 | 1.497 | -1.1 | -0.66 | .506 |
| **Healthcare accessibility** | 0.685 | 0.690 | -0.9 | -0.31 | .755 | | 0.611 | 0.612 | -0.3 | -0.16 | .872 |
| **Employment status** | 0.703 | 0.695 | 1.7 | 0.57 | .571 | | 0.710 | 0.700 | 2.2 | 1.31 | .189 |

**Table S3.** Balance test results for radius matching.

| **Variable** | **2018** | | | | | **2020** | | | | | |
| --- | --- | --- | --- | --- | --- | --- | --- | --- | --- | --- | --- |
|  | Mean | | % bias | T-test | | | Mean | | % bias | T-test | |
|  | Treated | Control |  | t | *P* > \| t \| | | Treated | Control |  | t | *P* > \| t |
| **Gender** | 0.449 | 0.444 | 0.9 | 0.29 | .773 | | 0.491 | 0.478 | 2.7 | 1.58 | .115 |
| **Age** | 55.566 | 55.403 | 1.9 | 0.76 | .446 | | 58.053 | 58.083 | -0.4 | -0.24 | .808 |
| **Residence** | 0.431 | 0.434 | -0.5 | -0.17 | .864 | | 0.513 | 0.507 | 1.3 | 0.73 | .465 |
| **Education** | 2.116 | 2.113 | 0.3 | 0.11 | .910 | | 1.740 | 1.758 | -2.1 | -1.18 | .237 |
| **Marital status** | 0.928 | 0.930 | -0.6 | -0.25 | .801 | | 0.917 | 0.908 | 2.7 | 1.87 | .061 |
| **Chronic disease** | 0.599 | 0.616 | -2.2 | -0.73 | .464 | | 0.471 | 0.504 | -4.7 | -2.75 | .006 |
| **Sleeping duration** | 1.402 | 1.427 | -1.9 | -0.69 | .490 | | 1.297 | 1.299 | -0.1 | -0.07 | .942 |
| **Napping duration** | 1.341 | 1.345 | -0.4 | -0.15 | .878 | | 1.376 | 1.338 | 3.5 | 2.07 | .038 |
| **Physical activity** | 1.535 | 1.541 | -0.8 | -0.28 | .776 | | 0.997 | 0.951 | 5.4 | 3.17 | .002 |
| **Drinking** | 0.515 | 0.498 | 3.5 | 1.15 | .252 | | 0.552 | 0.570 | -3.8 | -2.14 | .032 |
| **Household expenditure per capital** | 9.214 | 9.200 | 1.4 | 0.54 | .589 | | 9.127 | 9.116 | 1.1 | 0.73 | .463 |
| **Household size** | 2.830 | 2.832 | -0.1 | -0.04 | .964 | | 3.061 | 3.071 | -0.6 | -0.37 | .712 |
| **Household composition** | 1.455 | 1.457 | -0.3 | -0.10 | .917 | | 1.490 | 1.492 | -0.6 | -0.20 | .840 |
| **Healthcare accessibility** | 0.685 | 0.689 | -0.7 | -0.25 | .799 | | 0.611 | 0.611 | -0.1 | -0.05 | .958 |
| **Employment status** | 0.703 | 0.697 | 1.2 | 0.43 | .670 | | 0.710 | 0.697 | 2.9 | 1.71 | .088 |

**Table S4.** Balance test results for kernel matching.

| **Variable** | **2018** | | | | | **2020** | | | | | |
| --- | --- | --- | --- | --- | --- | --- | --- | --- | --- | --- | --- |
|  | Mean | | % bias | T-test | | | Mean | | % bias | T-test | |
|  | Treated | Control |  | t | *P* > \| t \| | | Treated | Control |  | t | *P* > \| t |
| **Gender** | 0.449 | 0.448 | 0.1 | 0.02 | .986 | | 0.491 | 0.484 | 1.4 | 0.85 | .397 |
| **Age** | 55.566 | 55.683 | -1.4 | -0.54 | .592 | | 58.053 | 58.177 | -1.5 | -1.03 | .304 |
| **Residence** | 0.431 | 0.442 | -2.3 | -0.72 | .473 | | 0.513 | 0.514 | -0.3 | -0.17 | .864 |
| **Education** | 2.116 | 2.086 | 3.5 | 1.19 | .234 | | 1.740 | 1.737 | 0.4 | 0.24 | .812 |
| **Marital status** | 0.928 | 0.927 | 0.4 | 0.14 | .891 | | 0.917 | 0.908 | 2.6 | 1.82 | .068 |
| **Chronic disease** | 0.599 | 0.614 | -2.0 | -0.67 | .506 | | 0.471 | 0.503 | -4.4 | -2.59 | .009 |
| **Sleeping duration** | 1.402 | 1.423 | -1.7 | -0.59 | .556 | | 1.297 | 1.300 | -0.1 | -0.07 | .945 |
| **Napping duration** | 1.341 | 1.343 | -0.2 | -0.09 | .932 | | 1.376 | 1.336 | 3.7 | 2.18 | .029 |
| **Physical activity** | 1.535 | 1.539 | -0.6 | -0.20 | .841 | | 0.997 | 0.955 | 4.9 | 2.92 | .004 |
| **Drinking** | 0.515 | 0.494 | 4.5 | 1.47 | .141 | | 0.552 | 0.576 | -5.0 | -2.85 | .004 |
| **Household expenditure per capital** | 9.214 | 9.177 | 3.6 | 1.40 | .162 | | 9.127 | 9.106 | 2.3 | 1.49 | .136 |
| **Household size** | 2.830 | 2.828 | 0.1 | 0.04 | .969 | | 3.061 | 3.077 | -1.0 | -0.60 | .548 |
| **Household composition** | 1.455 | 1.454 | 0.3 | 0.09 | .925 | | 1.490 | 1.490 | -0.1 | -0.03 | .973 |
| **Healthcare accessibility** | 0.685 | 0.685 | 0.1 | 0.04 | .965 | | 0.611 | 0.603 | 1.5 | 0.88 | .376 |
| **Employment status** | 0.703 | 0.695 | 1.6 | 0.55 | .580 | | 0.710 | 0.698 | 2.7 | 1.60 | .110 |

**Table S5.** Mediating effect of social participation between Internet use and depression using different matching methods.

| Matching methods | Types | β Coefficient (bootstrapped 95% CI ) | SE | *P* value |
| --- | --- | --- | --- | --- |
| K-nearest neighbor matching | **2018**  Indirect effect  Direct effect  Total effect  **2020**  Indirect effect  Direct effect  Total effect | -0.010 (-0.018 to -0.003)  -0.119 (-0.160 to -0.075)  -0.128 (-0.170 to -0.086)  -0.011 (-0.016 to -0.006)  -0.095 (-0.126 to -0.064)  -0.106 (-0.137 to -0.076) | 0.004  0.022  0.022  0.003  0.015  0.016 | .008  <.001  <.001  <.001  <.001  <.001 |
| Radius matching | **2018**  Indirect effect  Direct effect  Total effect  **2020**  Indirect effect  Direct effect  Total effect | -0.017 (-0.024 to -0.011)  -0.130 (-0.170 to -0.090)  -0.147 (-0.187 to -0.107)  -0.011 (-0.016 to -0.006)  -0.118 (-0.149 to -0.088)  -0.129 (-0.160 to -0.099) | 0.003  0.020  0.020  0.002  0.016  0.015 | <.001  <.001  <.001  <.001  <.001  <.001 |
| Kernel matching | **2018**  Indirect effect  Direct effect  Total effect  **2020**  Indirect effect  Direct effect  Total effect | -0.017 (-0.024 to -0.011)  -0.130 (-0.170 to -0.090)  -0.147 (-0.187 to -0.107)  -0.011 (-0.016 to -0.006)  -0.118 (-0.149 to -0.088)  -0.129 (-0.160 to -0.099) | 0.003  0.020  0.020  0.002  0.016  0.015 | <.001  <.001  <.001  <.001  <.001  <.001 |


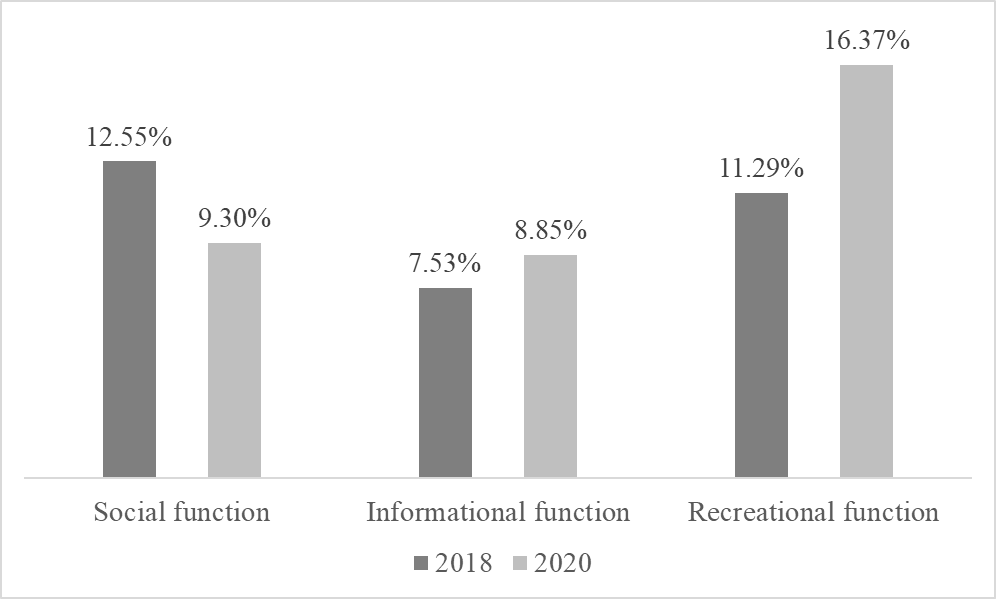


**Figure** S**1.** Comparison of proportions of mediating effects on the Internet use function.
